# Foliar spraying of exogenous uniconazole (S3307) at the flowering stage as an effective method to resist low-temperature stress on mung bean [*Vigna radiata* (L.) Wilczek]

**DOI:** 10.1038/s41598-023-49652-7

**Published:** 2023-12-15

**Authors:** Hongtao Xiang, Shiya Wang, Xiaoyan Liang, Xueyang Wang, Hongchang Xie, Deming Wang, Zhijia Gai, Nannan Wang, Peng Xiang, Dongwei Han, Dapeng Shan, Yichu Li, Wan Li

**Affiliations:** 1 Suihua Branch, Heilongjiang Academy of Agricultural Machinery Sciences, Suihua, 152054 Heilongjiang China; 2 Heilongjiang Academy of Agricultural Sciences, Harbin, 150086 Heilongjiang China; 3 College of Agriculture, Heilongjiang Bayi Agriculture University, Daqing, 163319 Heilongjiang China

**Keywords:** Physiology, Plant sciences

## Abstract

Low temperature is one of the major constraints on agricultural productivity worldwide and is likely to further increase. Several adaptations and mitigation strategies are required to cope with low-temperature stress. Uniconazole (S3307) could play a significant role in the alleviation of abiotic stress in plants. In this study, the effects of S3307 on the reactive oxygen species (ROS) and antioxidant metabolism were studied in the leaves of mung bean [*Vigna radiata* (L.) Wilczek]. The experimental results showed that the low-temperature induced accumulation of superoxide anion (O_2_^-^) production rate, and malonaldehyde (MDA) contents. Increased proline content and enzymatic antioxidants, including superoxide dismutase (SOD), catalase (CAT), and peroxidase (POD), were found to alleviate oxidative damage under low temperatures. While, S3307 could reduce O_2_^-^ production rate and MDA contents and increase the activities of SOD, POD, and CAT, slowed the decrease in ascorbic acid (AsA), dehydroascorbic acid (DHA), glutathione (GSH), and oxidized glutathione (GSSG), and promoted increase in soluble sugars (SS), soluble proteins (SP), and proline (Pro) content under low-temperature. At the same time, low temperature leads to lower 100 grain weight and number of grains per plant, which eventually causes yield reduction decreased. Foliar spraying of S3307 could alleviate the yield loss caused by low temperature, and the increase of S3307 treatment was 5.1%–12.5% and 6.3%–32.9% for the two varieties, respectively, compared with CK. In summary, exogenous S3307 pretreatment enhances plant tolerance to low-temperature by improving the antioxidant enzyme activities, increased non-enzymatic antioxidants content, and decreased O_2_^-^ production rate and MDA contents and inducing alterations in endogenous S3307, and reduce the decrease in mung bean yield.

## Introduction

Abiotic stresses reduce plant growth and production, posing significant risks to the global food supply. Plant growth and production might suffer as a result of low temperatures. Low-temperature stress is a prominent abiotic stress that causes significant crop loss worldwide. Plant stress responses are influenced by various factors, including exposure duration, species, and growth stage. Cold acclimation varies among plants native to different temperature regions, whereas 0–15 °C chilling temperature can be detrimental to many tropical and subtropical plants. According to Liu et al.^1^ and Nahar et al.^2^, low-temperature stress can alter plant membrane phase and permeability, leading to the overproduction of reactive oxygen species (ROS), which disrupts cellular homeostasis and metabolic integrity, and in severe cases, stunts plant growth and development and can even cause plant death.

Mung bean [*Vigna radiata* (L.) Wilczek], a valuable warm-season grain legume crop, is sensitive to low temperatures^3–5^. According to the Food and Agriculture Organization of the United Nations, global mung bean output increased fourfold between 1990 and 2014, reaching 21.4 million tons, with Asia accounting for 85.4% of total production. Because of their sensitivity to low temperatures, mung beans have a limited geographical and seasonal range. They thrive in subtropical regions of the world but are particularly vulnerable to temperature fluctuations. Despite being an economically significant crop, mung bean output is poor owing to abiotic and biotic stressors^6^. The initial flowering period is particularly sensitive to low temperatures because to the negative effects on reproductive activities, including pollen tube development, which is likely induced by low-temperature fertilization, resulting in lower yield^7^.

Under abiotic stress conditions, plant growth regulators play a vital role in regulating plant growth and stress resistance^8,9^. Uniconazole (S3307), a triazole plant growth regulator, is increasingly used to enhance plant growth. Excess ROS generation under adverse conditions can attack the plant membrane, causing lipid peroxidation and intensifying the damage. Application of S3307, by up-regulating non-enzymatic antioxidant content and antioxidant enzyme activity, can maintain normal plant growth while balancing ROS levels. Adverse stress results in a reduction in plant yield; however, spraying with S3307 can mitigate the losses to some extent. Therefore, S3307 is crucial to helping plants resist chilling stress.

Recent related studies have mainly focused on the regulatory mechanisms of antioxidant control during the seedling stage of bean as affected by low-temperature stress, while fewer studies have been conducted during the reproductive growth stage. Therefore, in this study, two mung bean varieties with different genotypes were selected and tested at the critical periods of nutritive and reproductive growth (flowering and R1 stage) to study and evaluate the effects of S3307 on antioxidant system, non-enzymatic antioxidants, osmotic regulators and yield under low-temperature stress conditions. This will provide a theoretical basis for improving mung bean low temperature tolerance and breeding for high yield and quality.

## Materials and methods

### Experimental design

The experiment was conducted at the potted planting field of the Institute of Crop Cultivation and Tillage, Heilongjiang Academy of Agricultural Sciences (119°32’E, 34°30’N). Uniform *Vigna radiata* L., Wilczek (commonly known as mung bean) seeds were obtained from the China National Practical Bean Technology System. Different genotypes of mung bean varieties, Lüfeng 2 (L2) and Lüfeng 5 (L5), were selected as test materials. The selected seeds were sterilized using 5% NaClO for three minutes before being thoroughly washed with distilled water. Each plastic pot, with dimensions of 30 cm in diameter and 25 cm in height, was filled with approximately 16 kg of soil, and five selected seeds were planted in each pot. To ensure uniform growth, thinning at the R1 growth stage retained four seedlings per pot.

This experiment has four treatments, denoted as CK (spray water and normal temperature), T1 (spray S3307 and normal temperature), T3 (spray water and an average temperature of 12 °C), and T4 (spray S3307 and an average temperature of 12 °C). When plants grow to flowering stage (R1) conduct foliar spraying of S3307 (concentration of 20 mg·L^−1^ and a spraying rate of 225 L·hm^−2^), and the pots were entered into the artificial climate room with different low temperatures for chilling treatment. The duration is 1, 2, 3, 4, and 5 days. Each treatment was sampled separately, immediately placed in liquid nitrogen, and stored in a -80 °C refrigerator for the determination of physiological indicators. Harvest yield measurements were taken at the full maturity stage (R8).

### Determination of the O_2_^-^ production rate, H_2_O_2_ and MDA content

0.5 g of the samples were ground into homogenate in 10 mL phosphate buffer (0.05 mM PBS, pH 7.8), centrifuge at 4000 × g for 20 min. Supernatant were used for malonaldehyde (MDA) and superoxide anion (O_2_^-^) determination.

The MDA content was evaluated using the 2-thiobarbituric acid (TBA) method. Add 2 ml of 0.6% TBA to 1 ml of the supernatant, mix well, and boil in a 100 °C boiling water bath for 15 min, cool quickly with cold water. Then centrifuged at 4000 × g for 20 min. The supernatant was collected to obtain OD values at 450, 532, and 600 nm^10^.

The O_2_^-^ production rate was determined using the hydroxylamine oxidation method^11^. The 0.5 mL extract was obtained by adding 0.5 mL PBS (0.05 M, pH 7.8) and 1 mL 10 mM hydroxylamine hydrochloride solution, then shaken well and left to stand for about 1 h at 25 °C. For chlorophyll extraction, 2 mL ether was added to the solution, followed by 1 mL 7 mM p-amino benzene sulfonic acid and then 1 mL 7 mM α-naphthylamine, then mixed well, swirled, and left to stand for 20 min at 25 °C. The mixture was centrifuged at 3000 rpm for 3 min and the OD was determined at 530 nm.

The hydrogen peroxide (H_2_O_2_) content was determined by a modified version of the method by Liu et al.^12^ For this, 1 g of the sample was added to 10 mL of acetone for 30 min at 4 °C. The homogenate obtained was centrifuged at 3000 rpm for 20 min. Then, 0.5 mL of the supernatant was added to 0.2 mL of 20%(w/v) titanium tetrachloride (TiCl_4_) in hydrogen chloride (HCl) and 0.2 ml of 100% ammonia. After centrifugation at 3000 rpm for 10 min, the supernatant was discarded and the precipitate was dissolved in 3 ml of 1 M Sulfuric acid (H_2_SO_4_). The absorbance values of the derived titanium–peroxidase complex at 410 nm were recorded.

### Determination of the activities of SOD, POD, and CAT

0.5 g of frozen leaf samples were ground into homogenate in 10 ml of 50 mM phosphate buffer (pH 7.8) on an ice bath and centrifuged at 4 °C at 12,000 × g for 20 min. The supernatant was used to determine enzyme activity.

Superoxide dismutase (SOD) activity was determined by the nitrogen blue tetrazolium (NBT) method at 560 nm^13^.

Peroxidase (POD) was determined by the oxidation rate of guaiacol at 470 nm^14^.

Catalase (CAT) was determined by a decrease in absorbance per minute at 240 nm^15^.

### Non-enzyme antioxidant levels

About 1 g of leaf sample was ground in 5 mL of 5% metaphosphoric acid. The homogenate was then centrifuged at 8000 g for 15 min. The supernatant was collected for the determination of AsA-GSH cycle products and substrate content. AsA (ascorbic acid) and DHA (dehydroascorbate) were determined using the method described by Law et al.^16^.

The contents of reduced GSH (glutathione) and GSSG (glutathione) were determined using the dithionitrobenzoic acid method by Rao et al.^17^.

### Determination of the content of osmoregulatory substances

0.5 g of the leaf sample, grind it with ethanol, centrifuge at 4 °C (12000 g × g for 10 min) and transfer it to a centrifuge tube. The above steps were repeated four times and then fixed to 25 mL with 80% ethanol solution. This ethanol extract was used for the determination of soluble sugar content. The soluble sugar content was determined by the anthrone colorimetric method at 620 nm^18^.

The soluble protein extraction method was the same as the antioxidant enzyme method. The colorimetric method was used for the determination^19^.

About 0.5 g of leaves sample, add 5 ml of 3% sulfosalicylic acid solution, extract in boiling water bath for 10 min and then filter, that is the extract of proline. Its content was determined using by the colorimetric method using ninhydrin^20^.

### Determination of yield and yield components

At the R8 stage, 10 plants were randomly selected from each treatment, and the pod number per plant, grain number per plant, and 100-seed weight were measured.

### Statistical analysis

Microsoft Excel 2013, and SPSS 25.0 were used to analyze the one-way ANOVA of all the collected data. Duncan test (*p* < 0.05) was used to evaluate the difference within treatments and the significant differences among different materials were determined.

### Ethical statement

We state that our experimental research on mung bean complies with the relevant institutional, national, and international guidelines and legislation.

## Results

### Membrane damage and ROS accumulation

**Figure 1:**
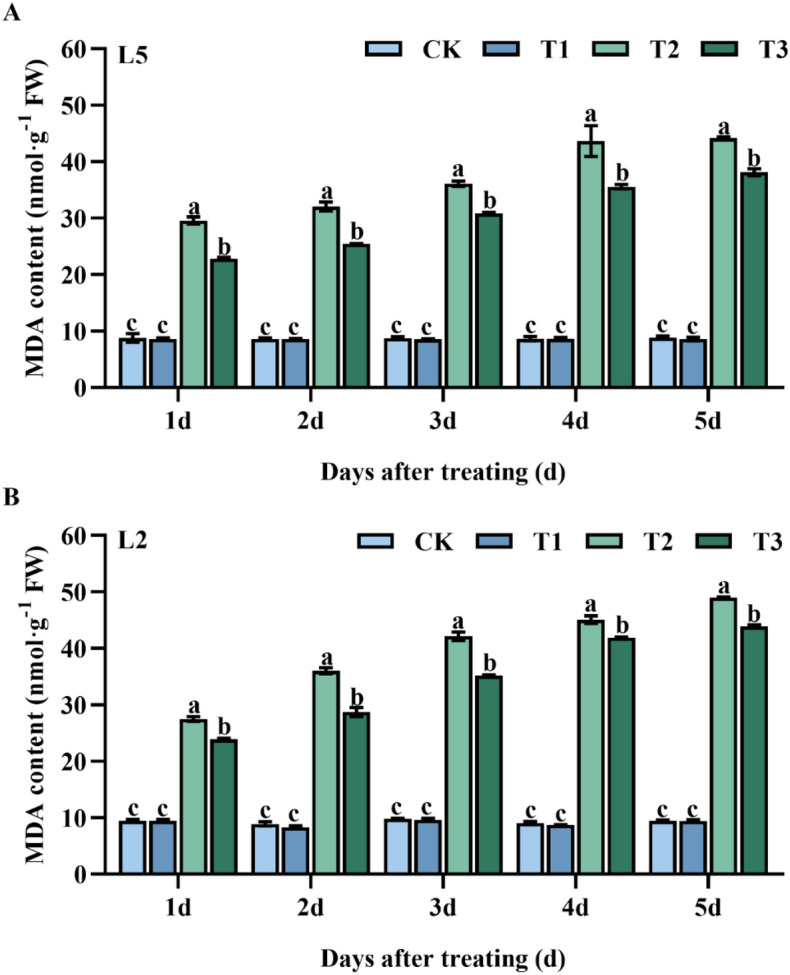
Effect of spraying exogenous S3307 on MDA content of mung beans leaves under chilling stress at flowering stage; CK, plant grown in natural environment + spraying H_2_O; T1, plant grown in natural environment + spraying S3307; T2, plant grown in Average 12 °C + spraying H_2_O; T3, plant grown in Average 12 °C + spraying exogenous S3307.

The results of this study demonstrated that spraying mung bean leaves with S3307 had a negligible effect on MDA content at normal temperatures (Fig. 1). No significant differences were observed between the treatment groups and the control for L5 and L2 at each sampling interval (T1). However, exposure to low temperatures caused a rapid increase in MDA content, with L5 and L2 exhibited considerably higher values compared to the control group at each sampling point during the T2 treatment. The application of S3307 was found to be an effective strategy for reducing MDA content under low-temperature conditions. Specifically, compared to T2, L5 showed reductions of 29.6, 26.1, 17.2, 22.8 and, 15.9% in T3 on 1–5 d of the treatment period, respectively, while L2 was reduced by 15.1, 25.4, 19.8, 7.7 and, 11.5%, respectively.

**Figure 2:**
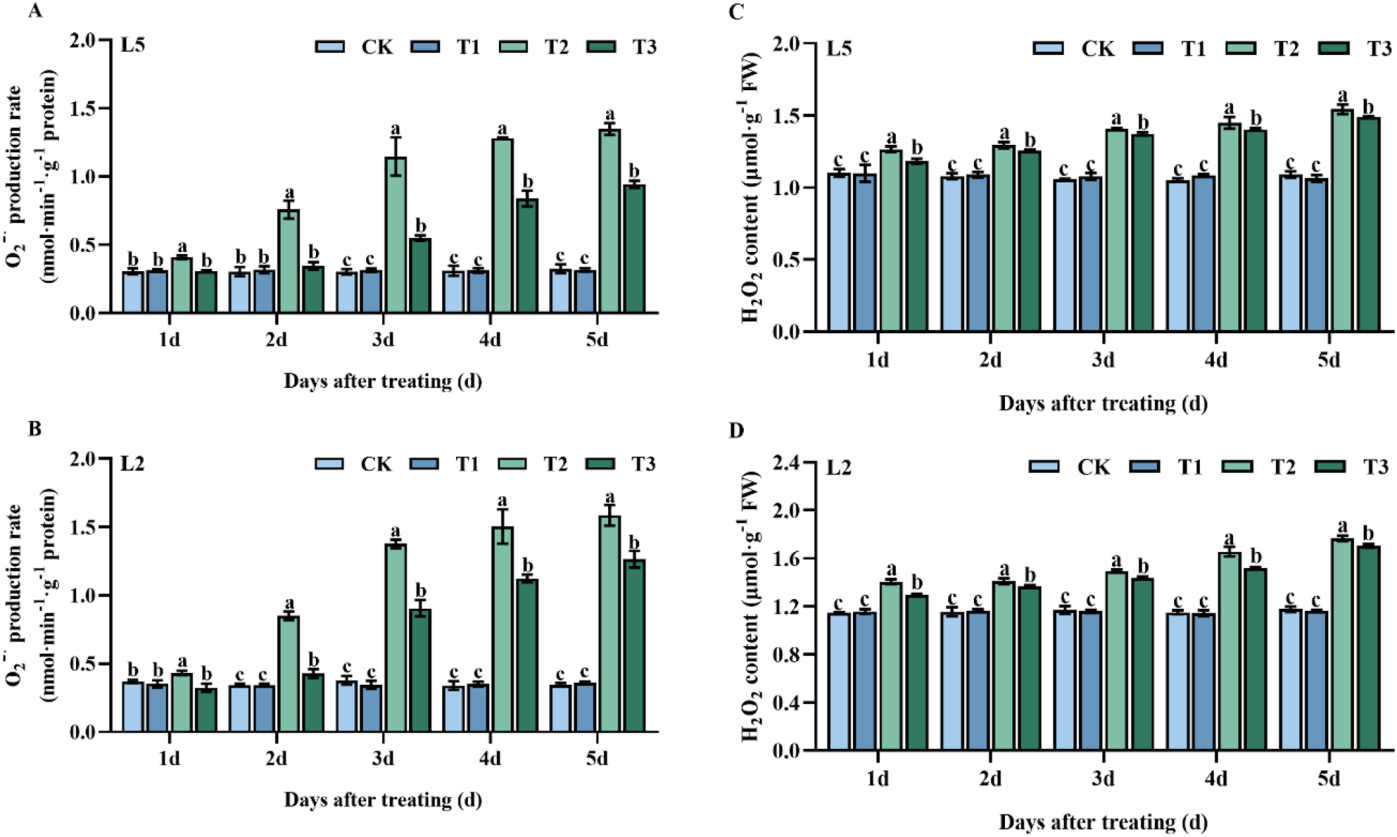
Effect of spraying exogenous S3307 on O_2_—production rate and H_2_O_2_ content of mung beans leaves under chilling stress at flowering stage; CK, plant grown in natural environment + spraying H_2_O; T1, plant grown in natural environment + spraying S3307; T2, plant grown in Average 12 °C + spraying H_2_O; T3, plant grown in Average 12 °C + spraying exogenous S3307.

Under chilling stress, O_2_^-^ and H_2_O_2_ levels in mung bean leaves increased dramatically, reaching considerably higher levels in the T2 treatment than in the control groups for L5 and L2 at each sampling point (Fig. 2A,B). However, the exogenous application of S3307 was found to effectively reduce ROS levels. Specifically, L5 showed a significant reduction in O_2_^-^ of 33.1%, 119.4%, 108.7%, 52.8%, and 42.9% during the T3 treatment compared to T2 at 1–5 d of treatment, respectively. Similarly, in the T3 treatment, L2 was significantly reduced by 33.3, 98.7, 52.0, 33.9 and, 25.5%, and 25.5% compared to T2 at days 1–5 of treatment, respectively. The trend for H_2_O_2_ was consistent with the trend for O_2_^-^ where L5 was reduced by 6.9, 3.0, 2.7, 3.2 and, 3.6% in T3 compared to T2 at 1–5 d of treatment, respectively. Likewise, L2 was reduced by 8.5, 3.1, 4.0, 9.0 and, 3.6% in T3 compared to T2 at 1–5 d of treatment, respectively (Fig. 2C,D).

**Figure 3:**
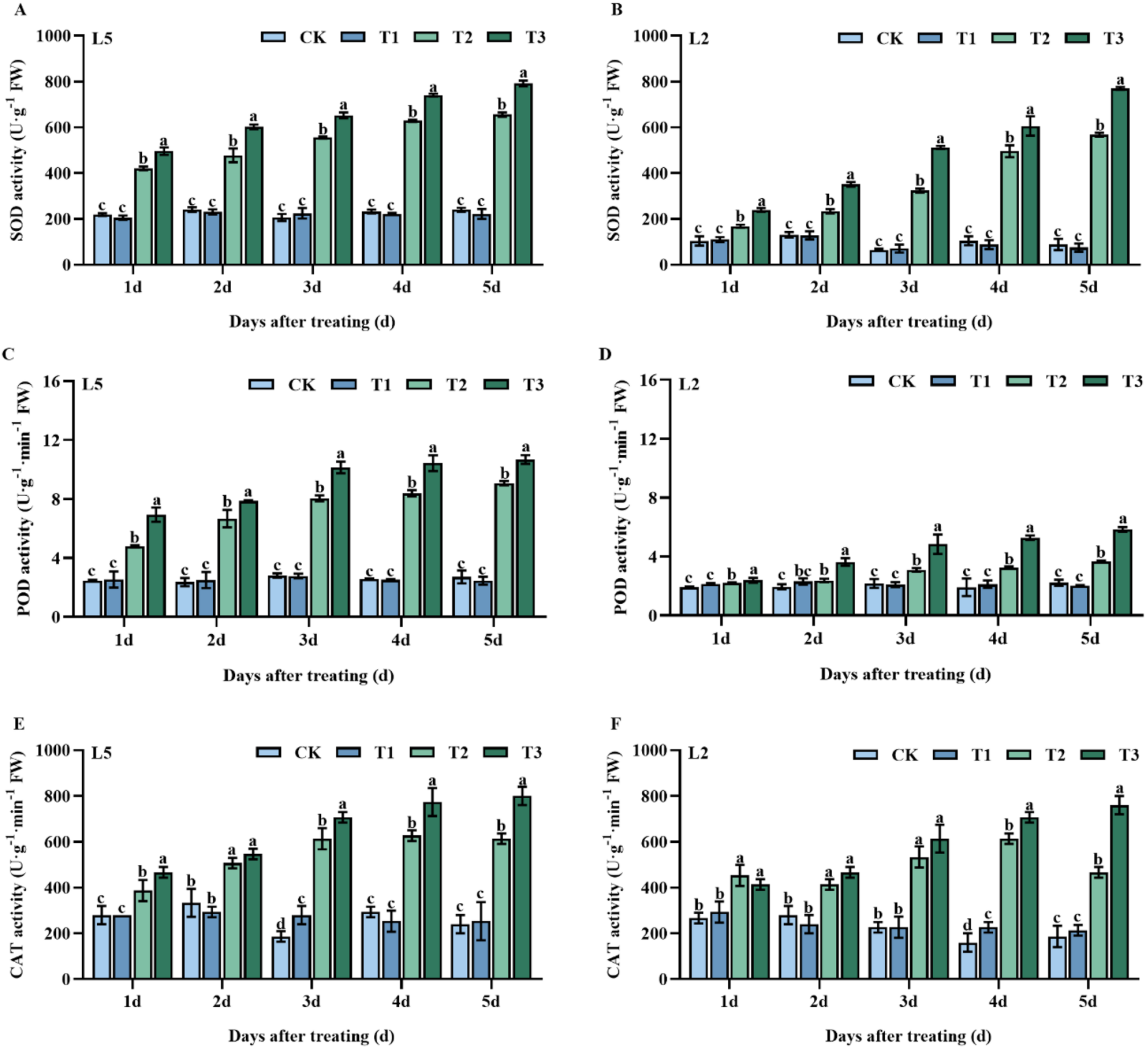
Effect of spraying exogenous S3307 on enzymatic antioxidant system of mung beans leaves under chilling stress at flowering stage; CK, plant grown in natural environment + spraying H_2_O; T1, plant grown in natural environment + spraying S3307; T2, plant grown in Average 12 °C + spraying H_2_O; T3, plant grown in Average 12 °C + spraying exogenous S3307.

The findings of this study illustrated that pre-spraying mung bean leaves with S3307 significantly increased CAT activity in L5 and L2 leaves at 3 d and 4 d, respectively. The increase was notable, with significant elevations of 50.0% and 42.0% for L5 T1 and L2 T1 treatments, respectively, as compared to the control group (CK) (Fig. 3C). Conversely, pre-spraying S3307 did not have any significant effect on the SOD and POD activities of either mung bean variety (Fig. 3A,B). Low-temperature treatment resulted in a rise in the activities of all three antioxidant enzymes, SOD, POD, and CAT. Foliar spraying of S3307 further activated these enzymes and led to greater enzyme activity in the cold-tolerant variety (L5) as compared to the cold-sensitive variety (L2). The activities of SOD, POD, and CAT were notably higher in both types of mung beans, as compared to CK, regardless of treatment duration (Fig. 3B,D,E,F).

There were highly significant effects of different varieties, different treatments and different sampling times on MDA, ROS system and antioxidant indexes (Table 1). From the interaction analysis between the two factors, it can be seen that the interaction effects between different varieties and different treatments (A × B), different varieties and different sampling times (A × C), and different treatments and different sampling times (B × C) had highly significant modulatory effects on MDA, O_2_^-^, H_2_O_2_, SOD, and POD activities, and that the interaction effects between different treatments and different sampling times (B × C) had highly significant effects on CAT activity. The interaction effect between different treatments and different sampling times (B × C) had a highly significant effect on CAT activity, while the other two factors had no significant effect on CAT. The interactions among the three factors showed that the interactions among different varieties, treatments and sampling times (A × B × C) had significant or highly significant regulatory effects on MDA, ROS system and antioxidant indexes. The above results indicate that low-temperature stress exacerbates cell membrane lipid peroxidation and ROS accumulation, while S3307 can alleviate the damage caused by low-temperature stress on leaves by increasing the activity of antioxidant enzymes.

**Table 1:** Variance analysis of leaf membrane damage and ROS accumulation under different varieties, different treatments, and different sampling time (*F* value).

Intercropping factor	MDA	O_2_^-^	H_2_O_2_	SOD	POD	CAT
Variety (A)	384.1**	208.6**	742.7**	2879.3**	2260.6**	48.9**
Treatment (B)	1025.6**	496.1**	223.0**	518.7**	131.7**	28.8**
Sampling time (C)	22,291.1**	2002.2**	2138.9**	5549.9**	1693.3**	756.0**
A × B	26.4**	12.282**	10.7**	28.1**	10.6**	3.9
A × C	71.5**	30.5**	21.6**	27.9**	501.2**	1.1
B × C	339.5**	178.1**	81.9**	199.8**	41.1**	32.6**
A × B × C	14.4**	4.9**	4.1**	24.0**	5.3**	2.8*

A, variety; B, treatment; C, sampling time; A × B, variety × treatment; A × C, variety × sampling time; B × C, treatment × sampling time; A × B × C, variety × treatment × sampling time; **, *p* < 0.05; *, *p* < 0.01.

### Effect of S3307 on AsA–GSH cycle none-enzymatic antioxidants in mung bean leaves under low temperature stress

**Figure 4:**
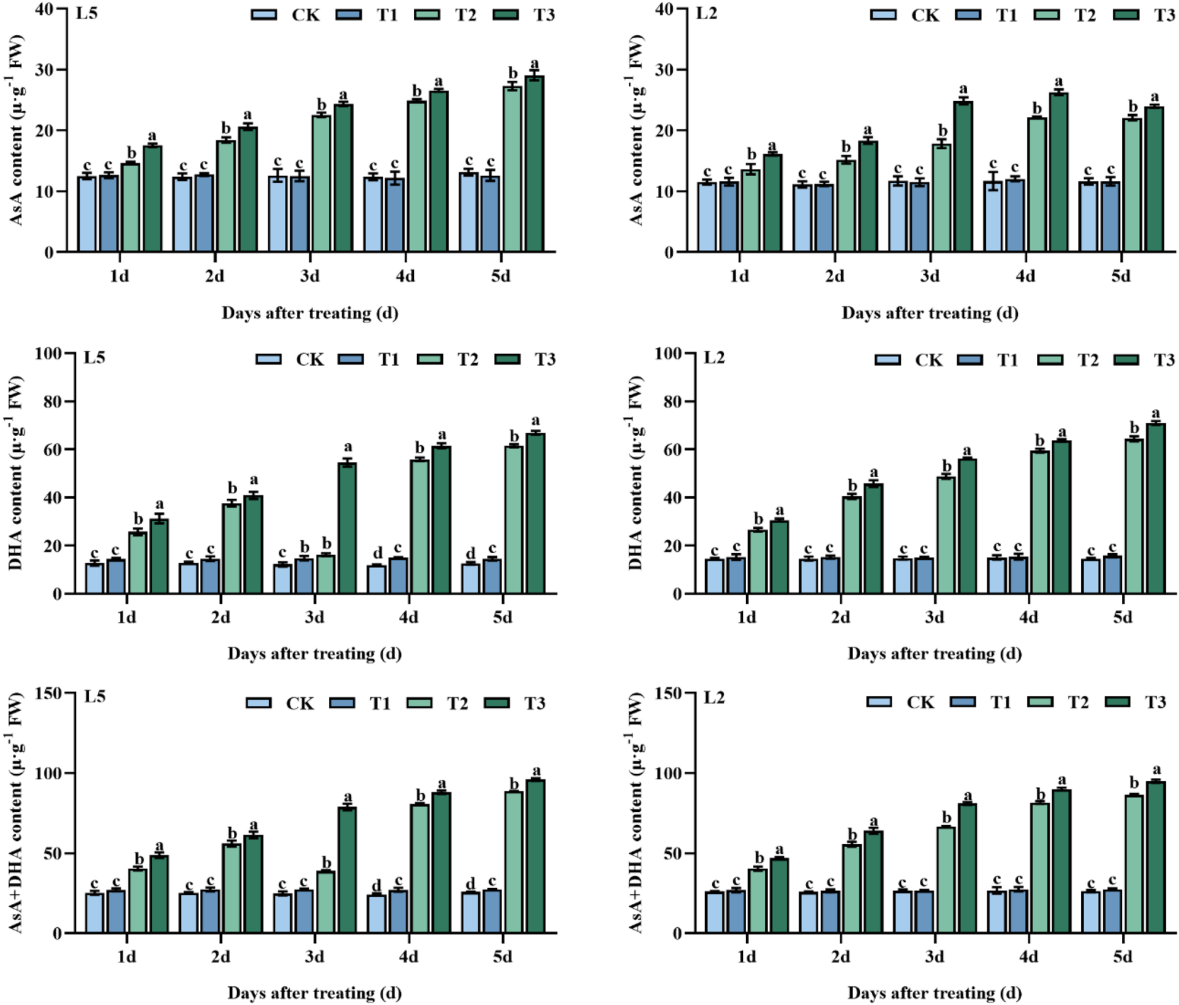
Effect of spraying exogenous S3307 on ascorbic acid content of mung beans leaves under chilling stress at flowering stage; CK, plant grown in natural environment + spraying H_2_O; T1, plant grown in natural environment + spraying S3307; T2, plant grown in Average 12 °C + spraying H_2_O; T3, plant grown in Average 12 °C + spraying exogenous S3307.

The data presented in Fig. 4 illustrates that spraying S3307 had a regulatory effect on DHA content, significantly increasing DHA content at 3–5 d relative to CK in T1 treatment by 21.4, 26.4 and, 15.6%, respectively, followed by an increase in both AsA + DHA content at 4 d and 5 d relative to CK by 12.0% and 6.0%, respectively. Pre-spraying S3307 had minimal to no impact on the levels of AsA, DHA, and AsA + DHA in L2 leaves. Both AsA and DHA contents in the leaves of both mung bean varieties significantly increased during low-temperature stress, with greater increases at the start of stress as compared to CK. The foliar application of S3307 further increased the concentrations of AsA, DHA, and AsA + DHA, particularly the cold-tolerant cultivar L5 (Fig. 4).

**Figure 5:**
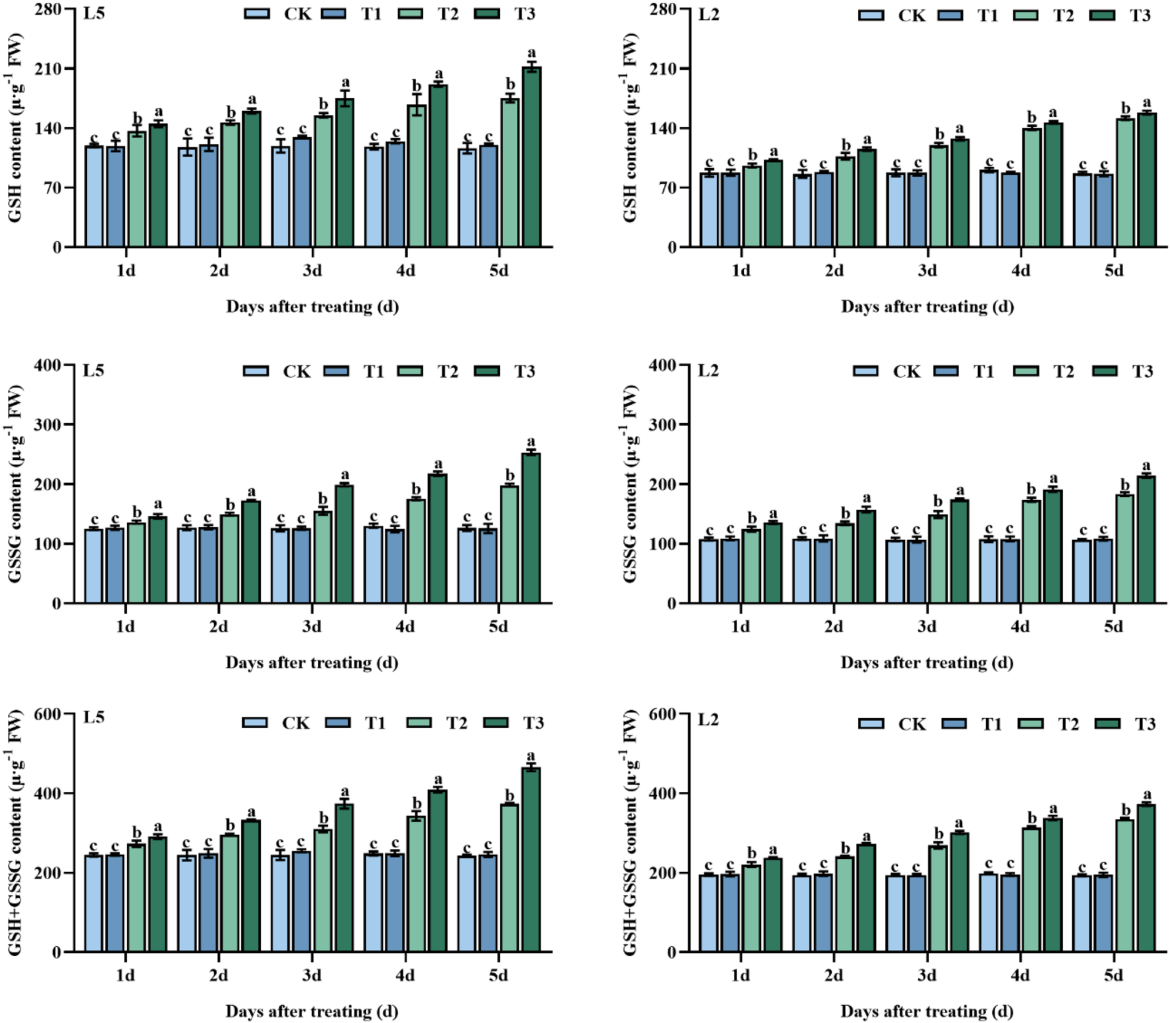
Effect of spraying exogenous S3307 on glutathione content of mung beans leaves under chilling stress at flowering stage; CK, plant grown in natural environment + spraying H_2_O; T1, plant grown in natural environment + spraying S3307; T2, plant grown in Average 12 °C + spraying H_2_O; T3, plant grown in Average 12 °C + spraying exogenous S3307.

The results of the study indicated that pre-spraying with S3307 did not significantly affect the GSH, GSSG, and GSH + GSSG content at ambient temperatures. However, at low-temperature conditions (T2 treatment), the content of these substances in both mung bean varieties showed a significant increase and the application of S3307 further enhanced the content of these indicators to a significant difference level. Additionally, the effect of spraying S3307 on GSH, GSSG, and GSH + GSSG content in L5 was greater than that of L2 under low-temperature stress (Fig. 5).

As shown in Table 2, there were highly significant effects of different varieties, different treatments and different sampling times on AsA, DHA and AsA + DHA contents. Meanwhile, two-factor and three-factor interaction effect analyses showed that there was a highly significant moderating effect on AsA, DHA and AsA + DHA contents by the interaction effects between different varieties and different treatments (A × B), different varieties and different sampling times (A × C), different treatments and different sampling times (B × C) and different varieties, different treatments and different sampling times (A × B × C).

**Table 2:** Variance analysis of leaf AsA-GSH cycle under different varieties, different treatments, and different sampling time (*F* value).

Intercropping factor	AsA	DHA	AsA + DHA	GSH	GSSG	GSH + GSSG
Variety (A)	248.5**	214.5**	1432.1**	68.1**	611.3**	4.5
Treatment (B)	313.6**	1536.9**	1365.2**	3.9	351.9**	118.5**
Sampling time (C)	2353.6**	14,006.5**	628.8**	15.1**	2237.9**	1172.7**
A × B	12.7**	10.7**	1139.1**	0.1	3.6	442.9**
A × C	24.5**	21.6**	167.0**	0.2	15.2**	143.8**
B × C	99.5**	520.8**	161.9**	0.9	130.8**	82.3**
A × B × C	6.1**	11.2**	127.2**	0.1	4.0**	117.6**

A, variety; B, treatment; C, sampling time; A × B, variety × treatment; A × C, variety × sampling time; B × C, treatment × sampling time; A × B × C, variety × treatment × sampling time; **, *p* < 0.05; *, *p* < 0.01.

There were highly significant effects of different varieties on GSH and GSSG and no significant effect on GSH + GSSG. Different treatments had significant regulatory effects on GSSG and GSH + GSSH and no significant effect on GSH. Different treatments had significant regulatory effects on GSH, GSSH and GSH + GSSH. Two-factor analysis showed that different varieties with different sampling times (A × C) and different treatments with different sampling times (B × C) had highly significant regulatory effects on GSSG and GSH + GSSG. Different varieties with different treatments (A × B) only had highly significant regulatory effects on GSH + GSSG. Three-factor analysis showed that the interaction effects between different varieties, different treatments and different sampling times (A × B × C) had highly significant regulatory effects on GSSG and GSH + GSSG. The above results suggest that different varieties, treatments and sampling times maintain the balance of the AsA-GSH cycle by increasing the AsA and DHA content and thus the AsA-GSH cycle.

### Effect of S3307 on the content of osmoregulatory substances in mung bean

**Figure 6:**
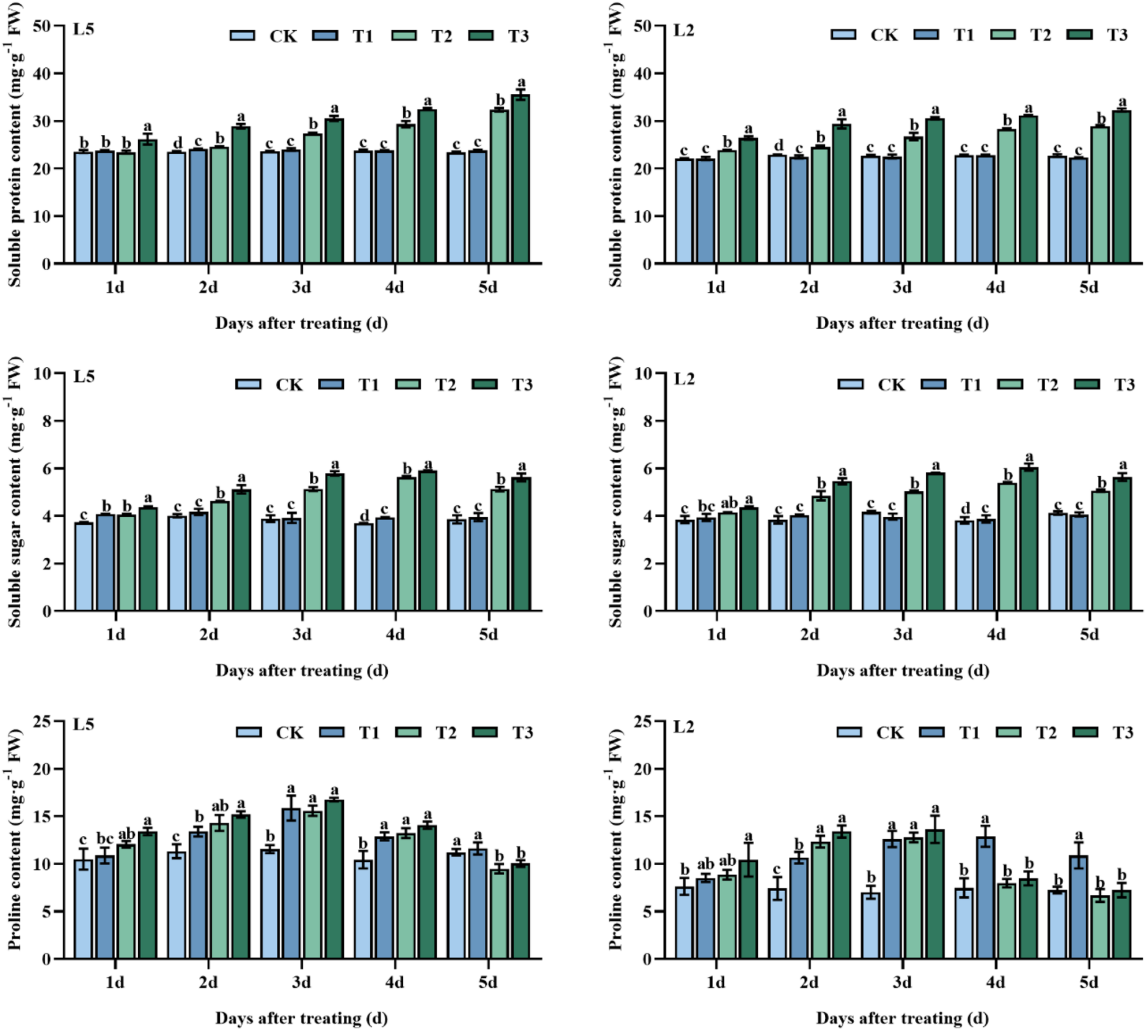
Effect of spraying exogenous S3307 on osmoregulation content of mung beans leaves under chilling stress at flowering stage; CK, plant grown in natural environment + spraying H_2_O; T1, plant grown in natural environment + spraying S3307; T2, plant grown in Average 12 °C + spraying H_2_O; T3, plant grown in Average 12 °C + spraying exogenous S3307.

During the low-temperature stress (T2), there was an increase in the soluble protein content of mung bean leaves, with L5 showing a significant increase of 4.6, 15.8, 23.3 and, 37.8% in T2 compared to CK at 2–5 d of treatment, and L2 showing a significant increase of 8.2, 7.2, 17.7, 24.0 and, 27.2% in T2 compared to CK at 1–5 d of treatment, respectively. Moreover, spraying S3307 under low-temperature conditions further enhanced the soluble protein content, with L5 showing a significant increase of 11.7, 17.3, 11.7, 10.8 and, 9.9%, respectively, in T3 treatment compared to T2 treatment at 1–5 d. Similarly, in T3 treatment, L2 showed a significant increase of 10.6, 19.8, 14.4, and, 10.2%, respectively, at 1–5 d compared to T2 treatment, with an overall increase of 11.7% (Fig. 6A,B).

Pre-treatment with S3307 had a moderating effect on the concentration of soluble sugars. Compared to the control group, all treatments administered between 1 and 5 d resulted in a significant increase in soluble sugar content in L5 leaves, with the most notable differences observed at 1 d and 4 d. However, pre-spraying with S3307 did not have a significant effect on L2 leaves. Low temperature caused an increase in soluble sugar content in mung bean leaves, with significant differences observed between the T2 treatment and the control group at all intervals except 2 d. Similarly, treatment with S3307 under low-temperature conditions led to a further increase in soluble sugar content of both varieties, with the T3 treatment producing a significantly higher concentration of soluble sugars compared to the T2 treatment (Fig. 6C,D).

Pre-treatment with S3307 under normal temperature conditions had varying effects on the proline content in the leaves of both mung bean varieties. The T1 treatment administered over 2–4 d resulted in significant increases of 18.3%, 37.3%, and 23.6% for the two varieties, respectively, compared to the control group (CK). However, other treatment times did not have a significant effect. For L2 leaves at 2–5 d, the T1 treatment significantly increased proline content by 43.8, 79.7, 72.7 and, 50.2%, respectively, compared to CK. Low-temperature stress increased the proline content in both leaves. In L5 leaves, the T2 treatment produced a significant increase of 14.9% and 26.2% at 1 d and 2 d of treatment, respectively, compared to CK. In L2 leaves, the T2 treatment resulted in a significant increase of 79.7% at 2 d of treatment compared to other treatment times and CK. Treatment with S3307 under low-temperature conditions mitigated the inhibition of proline content by low-temperature stress, resulting in an increase in both varieties under T3 treatment. However, the results did not reach a significant difference level (Fig. 6E,F).

As shown in Table 3, different varieties showed highly significant effect on soluble protein and proline content and no significant effect on soluble sugar content. Different treatments and different sampling times had highly significant effects on soluble protein, soluble sugar and proline contents. Two-factor analysis showed that different varieties and different treatments (A × B) had highly significant effect on soluble protein content and no significant effect on soluble sugar and proline content. Different varieties and different sampling time (A × C) had highly significant effect on soluble protein and proline content and no significant effect on soluble sugar content. While different treatments and different sampling time (B × C) had highly significant effect on soluble protein, soluble sugar and proline content. The three-factor analysis showed that the interaction effects among different varieties, different treatments and different sampling times (A × B × C) had highly significant regulatory effects on soluble protein, soluble sugar and proline contents.

**Table 3:** Variance analysis of leaf osmoregulatory substances under different varieties, different treatments, and different sampling time (*F* value).

Intercropping factor	Soluble protein	Soluble sugars	Proline
Variety (A)	193.0**	4.4	429.8**
Treatment (B)	309.5**	166.4**	94.1**
Sampling time (C)	2178.4**	1327.0**	95.3**
A × B	18.0**	0.7	1.8
A × C	4.4**	4.1	7.8**
B × C	96.7**	60.9**	19.1**
A × B × C	8.5**	3.9**	4.2**

A, variety; B, treatment; C, sampling time; A × B, variety × treatment; A × C, variety × sampling time; B × C, treatment × sampling time; A × B × C, variety × treatment × sampling time; **, *p* < 0.05; *, *p* < 0.01.

### Effect of S3307 on the content of yield and yield components in mung bean

According to Table 4, low temperatures during the initial flowering stage of mung bean resulted in a decrease in its 100-seed weight. At L5, T2 was reduced significantly by 2.2, 4.0, 8.5, 13.6 and, 16.6%, respectively, compared to the control group (CK) during the 1–5 d period. Similarly, at L2, T2 was significantly reduced by 3.3, 5.7, 13.1, 17.9 and, 22.1%, respectively, compared to CK during the 1–5 d period. Treatment with S3307 before exposure to low-temperature stress effectively mitigated the decrease in the 100-grain weight of mung bean. Specifically, at L5, T3 saw a significant increase of 1.4, 1.5, 5.2, 5.0 and, 3.6% compared to T2 during the 1–5 d period. Similarly, at L2, T3 saw a significant increase of 2.8, 3.2, 3.1, 4.8 and, 5.8% compared to T2 during the 1–5 d period (Table 4).

**Table 4:** Effect of S3307 on 100-seed weight of mung bean under low temperature stress.

Varieties	Treatment code	1 d	2 d	3 d	4 d	5 d
L5	CK	5.06 ± 0.02 a	5.06 ± 0.02 a	5.06 ± 0.03 a	5.06 ± 0.03 a	5.06 ± 0.01 a
	T1	5.11 ± 0.01 a	5.11 ± 0.01 a	5.11 ± 0.02 a	5.11 ± 0.02 a	5.11 ± 0.01 a
	T2	4.90 ± 0.02 c	4.82 ± 0.01 d	4.43 ± 0.05 c	4.22 ± 0.02 c	4.11 ± 0.01 c
	T3	4.97 ± 0.01 b	4.89 ± 0.01 c	4.66 ± 0.02 b	4.43 ± 0.03 b	4.26 ± 0.03 b
L2	CK	4.20 ± 0.02 a	4.20 ± 0.02 a	4.20 ± 0.02 b	4.20 ± 0.02 a	4.20 ± 0.02 a
	T1	4.26 ± 0.01 a	4.26 ± 0.01 a	4.26 ± 0.01 a	4.26 ± 0.01 a	4.26 ± 0.01 a
	T2	3.95 ± 0.02 c	3.77 ± 0.02 c	3.60 ± 0.01 d	3.36 ± 0.04 c	3.10 ± 0.04 c
	T3	4.06 ± 0.03 b	3.89 ± 0.04 b	3.71 ± 0.01 c	3.52 ± 0.02 b	3.28 ± 0.01 b

CK, plant grown in natural environment + spraying H_2_O; T1, plant grown in natural environment + spraying S3307; T2, plant grown in Average 12 °C + spraying H_2_O; T3, plant grown in Average 12 °C + spraying exogenous S3307.

Low-temperature stress caused a reduction in the number of grains per plant in both L5 and L2 treatments compared to the control (CK) between 1 and 5 d of treatment. Pre-spraying S3307 during low-temperature stress further reduced the number of grains per plant. However, in T3 treatment the reduction was significantly lower than in T2 only on the 4th and 5th day of treatment. In L5, T3 increased by 10.3% and 12.9% on the 4 d and 5 d, respectively, compared to T2. Similarly, in L2, T3 resulted in an increase of 12.0% and 13.5% on the 4 d and 5 d in comparison to T2 (Table 5).

**Table 5:** Effect of S3307 on mung bean number of grains per plant.

Varieties	Treatment code	1 d	2 d	3 d	4 d	5 d
L5	CK	127.33 ± 1.56 a	127.33 ± 1.01 a	127.33 ± 1.21 a	127.33 ± 1.21 a	127.33 ± 1.52 a
	T1	131.67 ± 1.91 a	131.67 ± 2.19 a	131.67 ± 2.25 a	131.67 ± 2.25 a	131.67 ± 2.08 a
	T2	116.67 ± 1.15 b	107.33 ± 1.56 b	96.67 ± 1.02 b	87.33 ± 2.32 c	77.67 ± 2.00 c
	T3	118.00 ± 1.83 b	113.00 ± 2.98 b	100.33 ± 2.67 b	96.67 ± 1.67 b	87.67 ± 2.02 b
L2	CK	118.33 ± 2.43 ab	118.33 ± 2.31 a	118.33 ± 2.46 a	118.33 ± 2.49 a	118.33 ± 2.65 a
	T1	123.00 ± 3.12 a	123.00 ± 3.18 a	123.00 ± 3.14 a	123.00 ± 3.09 a	123.00 ± 2.96 a
	T2	106.67 ± 1.59 c	99.33 ± 1.00 b	91.00 ± 0.65 b	80.33 ± 0.73 c	71.67 ± 1.20 c
	T3	110.67 ± 1.64 bc	106.67 ± 1.86 b	95.00 ± 0.65 b	90.00 ± 1.09 b	81.33 ± 0.01 b

CK, plant grown in natural environment + spraying H_2_O; T1, plant grown in natural environment + spraying S3307; T2, plant grown in Average 12 °C + spraying H_2_O; T3, plant grown in Average 12 °C + spraying exogenous S3307.

Pre-spraying S3307 significantly increased the grain weight per plant of both mung bean varieties, with T1 showing significant differences compared to the control (CK). Low-temperature stress decreased the grain weight per plant, and the effect was greater with longer stress periods. Spraying S3307 during low-temperature stress increased the grain weight per plant, where L5 resulted in a significant increase of 5.3, 5.1, 10.9, 12.5 and, 12.5% in T3 treatment compared to T2 treatment during 1–5 d of treatment, while L2 resulted in an increase of 6.4, 6.3, 21.2, 23.6 and, 32.9% respectively. In summary, the reduction in grain weight was greater in L5 than L2, and the impact of spraying S3307 under low-temperature stress was also stronger in L5 than L2 (Table 6).

**Table 6:** Effect of S3307 on mung bean yield under low temperature stress.

Varieties	Treatment code	1 d	2 d	3 d	4 d	5 d
L5	CK	5.11 ± 0.03 b	5.11 ± 0.01 b	5.11 ± 0.04 b	5.11 ± 0.04 b	5.11 ± 0.05 b
	T1	5.35 ± 0.03 a	5.35 ± 0.03 a	5.35 ± 0.02 a	5.35 ± 0.03 a	5.35 ± 0.03 a
	T2	4.35 ± 0.03 d	4.11 ± 0.03 d	3.67 ± 0.10 d	3.03 ± 0.04 d	2.65 ± 0.05 d
	T3	4.58 ± 0.07 c	4.32 ± 0.01 c	4.07 ± 0.04 c	3.41 ± 0.09 c	2.98 ± 0.05 c
L2	CK	5.34 ± 0.06 b	5.34 ± 0.04 b	5.34 ± 0.08 b	5.34 ± 0.07 b	5.34 ± 0.06 b
	T1	5.76 ± 0.06 a	5.76 ± 0.06 a	5.76 ± 0.05 a	5.76 ± 0.053 a	5.76 ± 0.04 a
	T2	4.24 ± 0.07 d	3.97 ± 0.03 d	3.16 ± 0.05 d	2.88 ± 0.02 d	2.34 ± 0.01 d
	T3	4.51 ± 0.04 c	4.22 ± 0.07 c	3.83 ± 0.04 c	3.56 ± 0.03 c	3.11 ± 0.03 c

CK, plant grown in natural environment + spraying H_2_O; T1, plant grown in natural environment + spraying S3307; T2, plant grown in Average 12 °C + spraying H_2_O; T3, plant grown in Average 12 °C + spraying exogenous S3307.

## Discussion

MDA is a primary product of membrane lipid peroxidation, which leads to oxidative stress in plant cells and damage to the membrane system, ultimately resulting in oxidative injury^21^. A direct reflection of the degree of membrane lipid peroxidation and indicator of stress damage is the MDA level. ROS production and reaction are associated with increases in MDA due to cellular oxidative damage under low temperature, causing changes in plant physiological and cell membrane performance^22^. Our findings demonstrate that MDA, H_2_O_2_, and O_2_ levels were elevated under low-temperature stress, but decreased following S3307 pretreatment. The decrease in ROS accumulation can be attributed to the improvement in antioxidant defense systems as a result of S3307 pretreatment^23^. Furthermore, it has been reported that S3307 pretreatment regulates plant antioxidant systems, effectively scavenging excessive free radicals. Based on our results, it can be concluded that S3307 pretreatment enhances antioxidant systems and positively influences the antioxidant network, maintaining the redox balance.

ROS plays a crucial role in the growth and development of plants^24^. Under normal conditions, the concentration of ROS is low, and it acts mainly as a signaling molecule to regulate plant growth and development. However, under adverse conditions, ROS accumulation increases dramatically, disrupting the degree of membrane lipid peroxidation and inhibiting the normal growth and development of plants^25^. Our results demonstrate that low-temperature stress induced an excessive accumulation of O_2_^-^ and an increase in H_2_O_2_ content with prolonged stress duration. Thus, it is essential for plant cells to scavenge ROS during stressful conditions to prevent toxicity. Antioxidative defense systems, including enzymatic and non-enzymatic tools, can effectively detoxify ROS in cells, as reported by Wu et al.^26^. Antioxidant enzymes such as SOD, POD, and CAT act as a protective enzyme system, limiting the levels of free radicals and preventing damage while maintaining a balance between antioxidants and free radicals^27,28^. In our study, low-temperature stress induced an increase in SOD, POD, and CAT activities in the leaves of two mung bean varieties, indicating that the plant's antioxidant systems were activated. Nevertheless, O_2_^-^ and H_2_O_2_ contents showed an increase, implying that enhanced antioxidant enzyme activities under low-temperature stress could not effectively scavenge the excessively accumulated ROS. The ROS intensifies membrane lipid peroxidation, ruptures double bonds of unsaturated fatty acids on the cell membrane, and damages the stability of the cell membrane, resulting in low temperature-induced plant damage. This outcome aligns with previous studies conducted on corn and mung beans^21,23^. S3307 enhances plant protection against diverse abiotic stresses. After S3307 spray application during low-temperature stress, both mung bean varieties leave SOD, POD, and CAT activities further increased, while O_2_^-^ and H_2_O_2_ contents decreased significantly. The above results indicated that S3307 can improve the antioxidant efficiency of mung bean leaves under low temperature stress, inhibit ROS accumulation and weaken membrane lipid peroxidation damage, thus improving the ability of plants to resist low temperature stress^29^.

Non-enzymatic antioxidants and osmoprotectants, besides antioxidant enzymes, play critical roles in mitigating various types of abiotic stress. In the Ascorbate–glutathione (AsA-GSH) cycle system, Ascorbate (AsA) and Glutathione (GSH) act as vital antioxidant substances^30^. AsA reduces oxidative damage in plant cells by effectively scavenging ROS while GSH promotes the structural stability of membrane proteins by oxidizing to produce GSSG during the reaction. Consequently, the content of AsA and GSH serves as essential indicators of plant antioxidant status^31^. Our study showed a significant increase in AsA content with low-temperature induction. In addition, foliar spraying of S3307 further increased the AsA content, thereby alleviating the damage caused by low-temperature stress. S3307 accelerates the conversion of MDHA and DHA to AsA by enhancing plant MDHAR and DHAR activities, consequently expediting the AsA-GSH cycle^32^. Meanwhile, the GSH and GSSG content showed an increase with prolonged chilling stress. GSH and GSSG content in leaves increased after S3307 treatment under chilling stress. The increase in GSSG content would help GR reduce it to GSH, which was very important to maintain reduced GSH^33^.

Osmoregulation is an important mechanism by which plants adapt to adverse conditions and improve their own regulation. They achieve this adaptation by altering the content of osmoregulatory substances such as soluble sugars (SS), soluble proteins (SP), and proline (Pro)^34^. SP provides a binding layer for bound water within the cell, protecting the cell structure from dehydration damage^35^. Soluble sugars and soluble proteins also function as osmoprotectants against dehydration damage, while proline assists in maintaining cellular osmotic balance, reducing the freezing point of cellular solutions, and protecting protein molecules against oxidative stress and biofilm damage^36,37^. In this study, the content of SS, SP, and Pro in the leaves of mung bean plants under low-temperature stress increased significantly, indicating that hypoxia stimulated the synthesis of osmoregulatory substances, reducing cellular water loss and improving plant adaptability to low-temperature stress. However, prolonged stress led to a reduction in the content of these substances, intensifying the depletion of osmoregulatory substances and forcing plants to reduce energy metabolism to resist inundation stress. Following the foliar spraying of S3307, increases in osmoregulatory substances content in leaf cells maintained cellular osmotic balance, reduced cellular water loss, and significantly improved the adaptability of mung bean plants to low-temperature stress^38^.

Temperature response significantly impacts the flowering process of mung beans. Low temperature reduces the leaf sucrose content and hinders transport outside of it, resulting in insufficient assimilates to the flowering organ, massive flower abscission, and eventually yield reduction. This study established that the duration of low temperature negatively correlates with the number of grains per plant, leading to significant yield reduction. Conversely, foliar spraying of S3307 during low-temperature stress increases the number of grains per plant and grain weight per plant of mung bean, thus alleviating yield loss under low temperatures.

## Conclusion

We conducted an analysis of the physiological responses and yield of mung beans under low temperature stress. Low temperature stress led to an increase in O_2_^-^ production rate and H_2_O_2_ content, which in turn damage to the cell membranes. This was supported by a significant increase in the MDA content. Hence, the S3307 treatment alleviated O_2_^-^ production rate, H_2_O_2_ and MDA content. It also improved mung bean tolerance to low temperature stress. S3307 treatment also increased the SOD, POD, CAT levels, upregulated AsA, DHA, GSH and GSSG content. The O_2_^-^ production rate and H_2_O_2_ and MDA content were much lower in the treated than in the control leaves. Thus, the S3307 treatment improved ROS scavenging and inhibited membrane lipid peroxidation. Our study showed that S3307 treatment mitigated the damage caused by low temperature stress during the flowering stage of mung bean and reduced yield loss by increasing the antioxidant capacity of mung bean leaves, the non-enzymatic antioxidant system and promoting the increase in the content of osmoregulatory substances.

## Data Availability

All data generated or analyzed during this study are included in this published article.
